# Hybridization of the swarming and interior point algorithms to solve the Rabinovich–Fabrikant system

**DOI:** 10.1038/s41598-023-37466-6

**Published:** 2023-07-06

**Authors:** Zulqurnain Sabir, Salem Ben Said, Qasem Al-Mdallal

**Affiliations:** Department of Mathematical Sciences, College of Science, United Arab Emirates University, P. O. Box 15551, Al Ain, UAE

**Keywords:** Engineering, Mathematics and computing

## Abstract

In this study, a trustworthy swarming computing procedure is demonstrated for solving the nonlinear dynamics of the Rabinovich–Fabrikant system. The nonlinear system’s dynamic depends upon the three differential equations. The computational stochastic structure based on the artificial neural networks (ANNs) along with the optimization of global search swarming particle swarm optimization (PSO) and local interior point (IP) algorithms, i.e., ANNs-PSOIP is presented to solve the Rabinovich–Fabrikant system. An objective function based on the differential form of the model is optimized through the local and global search methods. The correctness of the ANNs-PSOIP scheme is observed through the performances of achieved and source solutions, while the negligible absolute error that is around 10^−05^–10^−07^ also represent the worth of the ANNs-PSOIP algorithm. Furthermore, the consistency of the ANNs-PSOIP scheme is examined by applying different statistical procedures to solve the Rabinovich–Fabrikant system.

## Introduction

A noteworthy Rabinovich–Fabrikant system based on the chaotic system was developed by the eminent scientists Rabinovich and Fabrikant. This is a condensed version of a nonlinear complex parabolic system that models various physical processes, like as wind waves on water, the hydrodynamic flows based on the Tollmien–Schlichting waves, Langmuir waves in plasma, concentration waves using the chemical reactions with diffusion. First, the model's design is implemented in a physical system, which represent the modulation inconsistency using the medium of dissipative non-equilibrium^1,2^. Currently, it has been acknowledged in the model’s extraordinarily high dynamics along with various physical features^3^. Notably, the Lorenz and other chaotic models are based on the nonlinearities of the second kind. Whereas the Rabinovich–Fabrikant system has the third kind of nonlinearities using some remarkable dynamics, that is “virtual” saddles combined with numerous chaotic charming characters with distinctive characteristics as well as mysterious chaotic fascinations^4–11^. The systems having numerous dynamics that can exhibit the chaotic nonlinearities. The chaotic transients are established in the model and the chaotic transients have influential consequences for experimentations. To mention few of them are radio maps^12^, circuits^13^, hydrodynamics^14^, Lorenz system^15^, neural networks^16^, and R¨ossler system^17^.

A significant challenge for intellectual researchers is provided by using the modelling based on the system of nonlinear equations and one of the Rabinovich–Fabrikant chaotic systems that comprises an ordinary three coupled differential equations using the pioneer work of M. Rabinovich and A. Fabrikant, given as^2,18^:

1 $$\left\{ {\begin{array}{*{20}l} {u^{\prime}(\theta ) = \left( {u^{2} (\theta ) + m(\theta ) - 1} \right)v(\theta ) + eu(\theta ),} \hfill & {u(0) = k_{1} ,} \hfill \\ {v^{\prime}(\theta ) = \left( { - u^{2} (\theta ) + 3m(\theta ) + 1} \right)u(\theta ) + ev(\theta ),} \hfill & {v(0) = k_{2} ,} \hfill \\ {m^{\prime}(\theta ) = - 2\left( {u(\theta )v(\theta ) + f} \right)m(\theta ),} \hfill & {m(0) = k_{3} ,} \hfill \\ \end{array} } \right.$$

The above form of the nonlinear system has a variety of applications in various disciplines of mathematics and physics. *k*_1,_ *k*_2_ and *k*_3_ are in the initial form of the conditions, where *e* and *f* indicate the real finite constant values based on the model’s evolution control.

The current research relates to the solutions of the Rabinovich–Fabrikant system using the computational stochastic artificial neural networks (ANNs) together with the global search swarming particle swarm optimization (PSO) and local interior point (IP) algorithms, i.e., ANNs-PSOIP. Recently, these stochastic performances have been represented to solve various nonlinear and stiff natured models^19–25^, some of them are automated rail-mounted gantry crane model^26^, heterogeneous-vehicle capacitated arc routing problem^27^, drone-assisted camera network^28^, triboelectric sensors for surface identification^29^, thermal explosion system^30,31^, traffic flow prediction^32^, biological kind of Leptospirosis system^33,34^, high-dimensional expensive problems^35^, food chain nonlinear differential systems^36–38^, vector machine parameter optimization algorithm^39^, functional kind of systems^40,41^, wireless-powered systems^42^, singular nature nonlinear models^43–46^ and many more^47–50^. To inspire of these stochastic applications, the authors took keen interest to perform the solutions of the Rabinovich–Fabrikant system through the swarming computational procedures. Some innovative features are itemized as:

- The numerical solutions of the Rabinovich–Fabrikant system are presented efficiently by applying the proposed ANNs along with the swarming computational procedure.
- The consistent, trustworthy, and steady outputs of this system authenticate the correctness of the designed ANNs together with a swarming computational scheme.
- The small calculate absolute error (AE) performs the accuracy of the ANNs together with the swarming computational approach.
- The authentication of the computational ANNs together with the swarming computational approach is established by taking three statistical operators with 50 executions to solve the model.

The rest of the presentation of the paper is given as: The procedure of the ANNs together with the swarming scheme is given in Section "Proposed ANNs-PSOIP method". The numerical solutions with different plots and tables are presented in Section "Result performances". The conclusions are drawn in the last Section.

## Proposed ANNs-PSOIP method

The Rabinovich–Fabrikant system is solved numerically by applying the swarming computational procedures. The mathematical neural network formulations are shown as:

2 $$\begin{gathered} [\hat{u}(\theta ),\hat{v}(\theta ),\hat{m}(\theta )] = \left[ {\sum\limits_{i = \,1}^{p} {q_{u,i} Y(w_{u,i} \theta + r_{u,i} ),} \,\sum\limits_{i = \,1}^{p} {q_{v,i} Y(w_{v,i} \theta + r_{v,i} ),\sum\limits_{i = \,1}^{p} {q_{m,i} Y(w_{m,i} \theta + r_{m,i} )} } } \right], \hfill \\ [\hat{u}^{\prime}(\theta ),\hat{v}^{\prime}(\theta ),\hat{m}^{\prime}(\theta )] = \left[ {\sum\limits_{i = \,1}^{p} {q_{u,i} Y^{\prime}(w_{u,i} \theta + r_{u,i} ),} \,\sum\limits_{i = \,1}^{p} {q_{v,i} Y^{\prime}(w_{v,i} \theta + r_{v,i} ),\sum\limits_{i = \,1}^{p} {q_{m,i} Y^{\prime}(w_{m,i} \theta + r_{m,i} )} } } \right], \hfill \\ \end{gathered}$$

where, *p, Y* present the neurons and activation function, while the unknown weights ***W***, shown as ${\varvec{W}} = [{\varvec{W}}_{u} ,\,{\varvec{W}}_{v} \user2{,W}_{m} ]$, for ${\varvec{W}}_{u} = [{\varvec{q}}_{u} \user2{,\omega }_{u} ,{\varvec{r}}_{u} ]$, ${\varvec{W}}_{v} = [{\varvec{q}}_{v} \user2{,\omega }_{v} ,{\varvec{r}}_{v} ]$, and ${\varvec{W}}_{m} = [{\varvec{q}}_{m} \user2{,\omega }_{m} ,{\varvec{r}}_{m} ]$, where

$$\begin{array}{*{20}l} {q_{u} = \left[ {q_{u,1} ,q_{u,2} , \ldots ,q_{u,p} ]} \right],} \hfill & {q_{v} = \left[ {q_{v,1} ,q_{v,2} , \ldots ,q_{v,p} } \right],} \hfill & {q_{m} = \left[ {q_{m,1} ,q_{m,2} , \ldots ,q_{m,p} } \right],} \hfill \\ {w_{u} = \left[ {w_{u,1} ,w_{u,2} , \ldots ,w_{u,p} } \right],} \hfill & {w_{v} = \left[ {w_{v,1} ,w_{v,2} , \ldots ,w_{v,p} } \right],} \hfill & {w_{m} = \left[ {w_{m,1} ,w_{m,2} , \ldots ,w_{m,p} } \right],} \hfill \\ {r_{u} = \left[ {r_{u,1} ,r_{u,2} , \ldots ,r_{u,p} } \right],} \hfill & {r_{v} = \left[ {r_{v,1} ,r_{,2v} , \ldots ,r_{v,p} } \right],} \hfill & {r_{m} = \left[ {r_{m,1} ,r_{m,2} , \ldots ,r_{m,p} } \right].} \hfill \\ \end{array}$$

The mathematical form of the transfer log-sigmoid function is given as: $Y(\theta ) = \left( {1 + e^{ - \theta } } \right)^{ - 1}$.

3 $$\begin{gathered} [\hat{u}(\theta ),\hat{v}(\theta ),\hat{m}(\theta )] = \left[ {\sum\limits_{i = 1}^{p} {\frac{{q_{u,i} }}{{1 + e^{{ - \left( {w_{u,i} \theta + r_{u,i} } \right)}} }},\sum\limits_{i = 1}^{p} {\frac{{q_{v,i} }}{{1 + e^{{ - \left( {w_{v,i} \theta + r_{v,i} } \right)}} }},\sum\limits_{i = 1}^{p} {\frac{{q_{m,i} }}{{1 + e^{{ - \left( {w_{m,i} \theta + r_{m,i} } \right)}} }}} } } } \right], \hfill \\ [\hat{u}^{\prime}(\theta ),\hat{v}^{\prime}(\theta ),\hat{m}^{\prime}(\theta )] = \left[ {\sum\limits_{i = 1}^{q} {\frac{{q_{u,i} w_{u,i} e^{{ - \left( {w_{u,i} \theta + r_{u,i} } \right)}} }}{{\left( {1 + e^{{ - \left( {w_{u,i} \theta + r_{u,i} } \right)}} } \right)^{2} }},\,\sum\limits_{i = 1}^{q} {\frac{{q_{v,i} w_{y,i} e^{{ - \left( {w_{v,i} \theta + r_{v,i} } \right)}} }}{{\left( {1 + e^{{ - \left( {w_{v,i} \theta + r_{v,i} } \right)}} } \right)^{2} }}} } ,\sum\limits_{i = 1}^{q} {\frac{{q_{m,i} w_{y,i} e^{{ - \left( {w_{m,i} \theta + r_{m,i} } \right)}} }}{{\left( {1 + e^{{ - \left( {w_{m,i} \theta + r_{m,i} } \right)}} } \right)^{2} }}} } \right]. \hfill \\ \end{gathered}$$

A merit function is designed as:

4 $$e = \sum\limits_{j = 1}^{4} {e_{j} }$$

5 $$e_{1} = \frac{1}{N}\sum\limits_{c = 1}^{N} {\left[ {\hat{u}^{\prime}_{c} - \left( {\hat{u}_{c}^{2} + \hat{m}_{c} - 1} \right)\hat{v}_{c} - e\hat{u}_{c} } \right]^{2} ,\,}$$

6 $$e_{2} = \frac{1}{N}\sum\limits_{c = 1}^{N} {\left[ {\hat{v}_{c} - \left( { - \hat{u}_{c}^{2} + 3\hat{m}_{c} + 1} \right)\hat{u}_{c} - e\hat{v}_{c} } \right]^{2} ,\,}$$

7 $$e_{3} = \frac{1}{N}\sum\limits_{c = 1}^{N} {\left[ {\hat{m}^{\prime}_{c} + 2\left( {f + \hat{u}_{c} \hat{v}_{c} } \right)\hat{m}_{c} } \right]^{2} ,\,}$$

8 $$e_{4} = \frac{1}{3}\left[ {\left( {\hat{u}_{0} - k_{1} } \right)^{2} + \left( {\hat{v}_{0} - k_{2} } \right)^{2} + \left( {\hat{m}_{0} - k_{3} } \right)^{2} } \right],$$

where $$\hat{u}_{c} = u(\theta_{c} ),\,\hat{v}_{c} = v(\theta_{c} ),\hat{m}_{c} = m(\theta_{c} ),\,Nh = 1,\,$$ and $$\,\theta_{c} = hc$$.

### Optimization schemes

The optimizations through the PSOIP for solving the Rabinovich–Fabrikant system are presented in this section.

PSO is a global search neuro swarming scheme introduced by Kennedy and Eberhart in the previous century, which works as an alteration of genetic algorithm (GA)^51^. PSO exhibits the outcomes of multiple intricate systems that manage a specific population through the technique of optimum training. PSO works by using the minimum storage capacity^52^. In recent decades, PSO is used in various submissions, like as mixed-variable optimization systems^53^, engineering networks^54^, multi-objective multimodal approaches^55^, solar form of the energy sets^56^, photovoltaic parameters category based on single, dual and three-ways diode^57^, studies of plant illnesses^58^, image recognition^59^, particle filter noise reduction based on mechanical accountability^60^, and production systems using the green coal^61^. These remarkable proposals motivated the authors to operate the swarming approaches for the Rabinovich–Fabrikant system.

**Figure 1:**
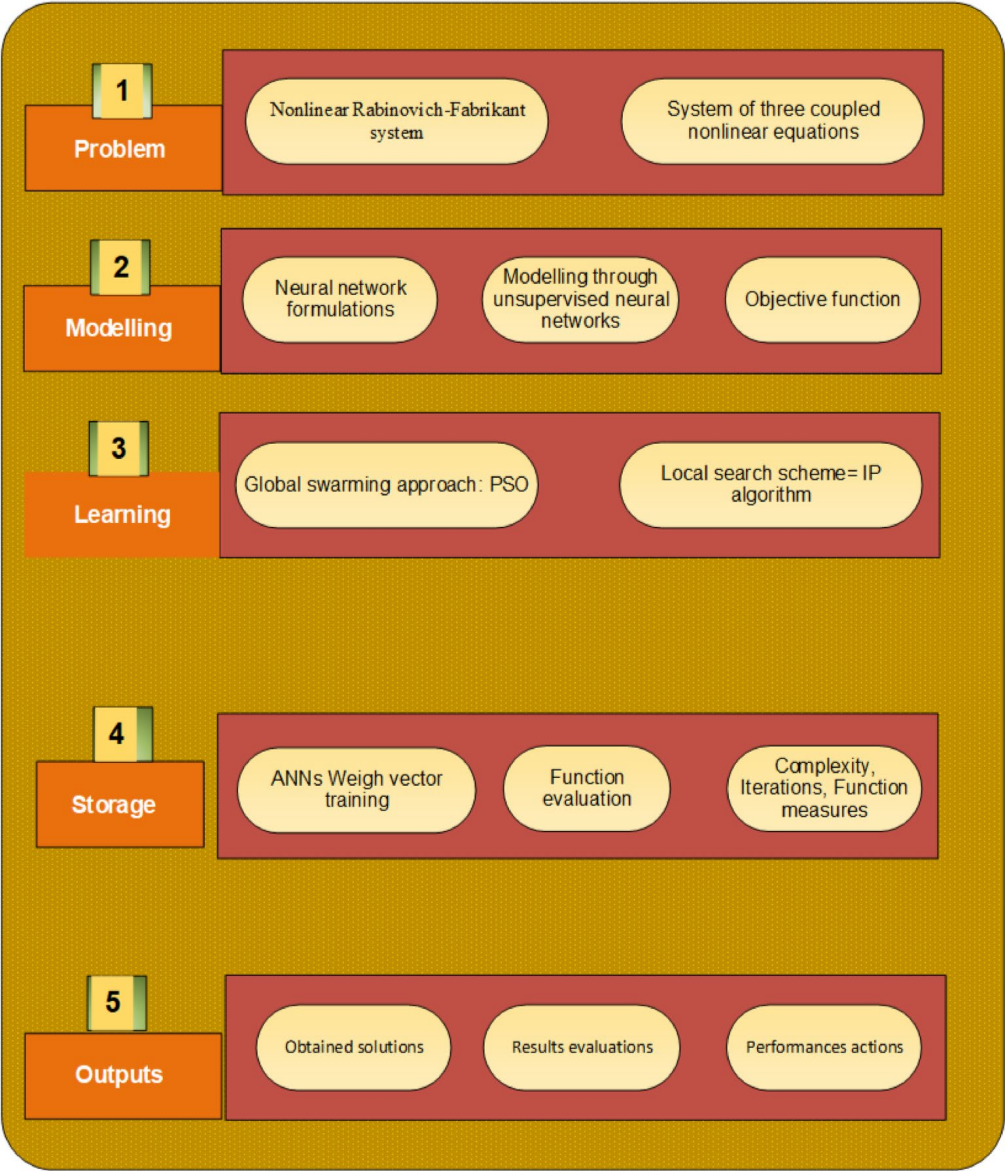
Designed swarming and local search procedures to solve the Rabinovich–Fabrikant system.

The global PSO process is considered a slow and sluggish scheme like the GA, which perform rapid convergence with the hybridization of the local search method. Consequently, IP approach is used by taking the primary inputs of the global PSO. IP is an outstanding scheme, which is applied to model the unconstrained/constrained systems. Some important IP applications are the shrinking horizon model predictive control with variable discretization step^62^, quantum key distribution rate computation^63^, parameters estimation using the symmetric spinning projectiles based on the maximum likelihood scheme^64^, equilibrium problems of the fisher market using the kernel function^65^, and symmetric cone horizontal linear complementarity model using the function of positive-asymptotic barrier^66^. The process of the swarming scheme along with a local search method to solve the Rabinovich–Fabrikant system is presented in Fig. 1.

### Statistical measures

In this section, the statistical performances based on the semi-interquartile range (SIR), mean square error (MSE), and Theil’s inequality coefficient (TIC) are shown as:

9 $$\left\{ \begin{gathered} {\text{SIR}} = 0.5\left( {Q_{3} - Q_{1} } \right), \hfill \\ Q_{3} \,\& Q_{1} = {\text{3rd}}\,\& 1{\text{st}}\,{\text{quartile,}}\, \hfill \\ \end{gathered} \right.$$

10 $${\text{[MSE}}_{u} ,{\text{MSE}}_{v} {\text{, MSE}}_{m} ] = \left[ {\sum\limits_{l = 1}^{m} {\left( {u_{l} - \hat{u}_{l} } \right)^{2} } ,\sum\limits_{l = 1}^{m} {\left( {v_{l} - \hat{v}_{l} } \right)^{2} } ,\sum\limits_{l = 1}^{m} {\left( {m_{l} - \hat{m}_{l} } \right)^{2} } } \right],$$

11 $$\left[ {{\text{TIC}}_{u} ,{\text{TIC}}_{v} ,{\text{TIC}}_{m} } \right]{ = }\left[ \begin{gathered} \frac{{\sqrt {\frac{1}{n}\sum\limits_{l = 1}^{n} {\left( {u_{l} - \hat{u}_{l} } \right)^{2} } } }}{{\left( {\sqrt {\frac{1}{n}\sum\limits_{l = 1}^{n} {u_{l}^{2} } } + \sqrt {\frac{1}{n}\sum\limits_{l = 1}^{n} {\hat{u}_{l}^{2} } } } \right)}},\frac{{\sqrt {\frac{1}{n}\sum\limits_{l = 1}^{n} {\left( {v_{l} - \hat{v}_{l} } \right)^{2} } } }}{{\left( {\sqrt {\frac{1}{n}\sum\limits_{l = 1}^{n} {v_{l}^{2} } } + \sqrt {\frac{1}{n}\sum\limits_{l = 1}^{n} {\hat{v}_{l}^{2} } } } \right)}}, \hfill \\ \frac{{\sqrt {\frac{1}{n}\sum\limits_{l = 1}^{n} {\left( {m_{l} - \hat{m}_{l} } \right)^{2} } } }}{{\left( {\sqrt {\frac{1}{n}\sum\limits_{l = 1}^{n} {m_{l}^{2} } } + \sqrt {\frac{1}{n}\sum\limits_{l = 1}^{n} {\hat{m}_{l}^{2} } } } \right)}} \hfill \\ \end{gathered} \right].$$

## Result performances

The numerical solutions of the Rabinovich–Fabrikant system (1) are presented by using the swarming computing procedures. The plots of results overlapping, weights, statistical performances along with the AE are also illustrated in this section. The system (1) is updated by using the suitable values as:

12 $$\left\{ {\begin{array}{*{20}l} {u^{\prime}(\theta ) = \left( {u^{2} (\theta ) + m(\theta ) - 1} \right)v(\theta ) + 2u(\theta ),} \hfill & {u(0) = 0.1,} \hfill \\ {v^{\prime}(\theta ) = \left( { - u^{2} (\theta ) + 3m(\theta ) + 1} \right)u(\theta ) + 2v(\theta ),} \hfill & {v(0) = 0.2,} \hfill \\ {m^{\prime}(\theta ) = - 2\left( {u(\theta )v(\theta ) + 3} \right)m(\theta ),} \hfill & {m(0) = 0.3.} \hfill \\ \end{array} } \right.$$

A fitness function is shown as:

13 $$\begin{gathered} e = \frac{1}{N}\sum\limits_{c = 1}^{N} {\left( \begin{gathered} \left[ {\hat{u}^{\prime}_{c} - \left( {\hat{u}_{c}^{2} + \hat{m}_{c} - 1} \right)\hat{v}_{c} - e\hat{u}_{c} } \right]^{2} + \left[ {\hat{v}_{c} - \left( { - \hat{u}_{c}^{2} + 3\hat{m}_{c} + 1} \right)\hat{u}_{c} - e\hat{v}_{c} } \right]^{2} \hfill \\ + \left[ {\hat{m}^{\prime}_{c} + 2\left( {f + \hat{u}_{c} \hat{v}_{c} } \right)\hat{m}_{c} } \right]^{2} \hfill \\ \end{gathered} \right)} \hfill \\ \,\,\,\,\,\, + \frac{1}{3}\left[ {\left( {\hat{u}_{0} - 0.1} \right)^{2} + \left( {\hat{v}_{0} - 0.2} \right)^{2} + (\hat{m}_{0} - 0.3)^{2} } \right]. \hfill \\ \end{gathered}$$

The optimization of objective function given in system (13) is provided by using the ANNs together with the swarming computational approach to solve the Rabinovich–Fabrikant system. Ten numbers of neurons along with 50 runs have been executed to check the reliability of the procedure. The optimal results based on the weight vectors for solving the above system are shown below.

14 $$\begin{gathered} \hat{u}(\theta ) = \frac{0.0042}{{1 + e^{ - (7.3325\theta - 5.1850)} }} - \frac{5.5634}{{1 + e^{ - ( - 13.460\theta + 19.964)} }} - \frac{0.3437}{{1 + e^{ - ( 2.8283\theta - 1.7191)} }} - \frac{5.6722}{{1 + e^{ - ( 1.1198\theta - 10.419)} }} \hfill \\ \,\,\,\,\,\,\,\,\,\,\, + \frac{0.1638}{{1 + e^{ - ( 3.7726\theta + 2.1030)} }} + \frac{2.5293}{{1 + e^{ - (5. - 277\theta - 6.3198)} }} - \frac{0.2724}{{1 + e^{ - ( 21.008\theta - 26.939)} }} + \frac{5.4190}{{1 + e^{ - ( - 4.135\theta + 5.0166)} }} \hfill \\ \,\,\,\,\,\,\,\,\,\,\,\, + \frac{0.9113}{{1 + e^{ - ( 1.7256\theta - 1.3888)} }} - \frac{2.5793}{{1 + e^{ - (4.3167\theta - 11.933)} }}, \hfill \\ \end{gathered}$$

15 $$\begin{gathered} \hat{v}(\theta ) = \frac{13.6431}{{1 + e^{ - (10.727\theta + 20.281)} }} - \frac{0.0229}{{1 + e^{ - (1.5494\theta + 5.9631)} }} - \frac{5.8231}{{1 + e^{ - ( - 6.3142\theta + 9.5120)} }} - \frac{5.0170}{{1 + e^{ - ( 0.316\theta + 3.209)} }} \hfill \\ \,\,\,\,\,\,\,\,\,\,\, - \frac{2.3954}{{1 + e^{ - ( - 0.437\theta + 0.2973)} }} - \frac{1.4387}{{1 + e^{ - ( - 3.303\theta + 2.755)} }} - \frac{7.3507}{{1 + e^{ - ( - 7.8204\theta - 9.816)} }} + \frac{2.3850}{{1 + e^{ - ( 0.8992\theta - 9.8543)} }} \hfill \\ \,\,\,\,\,\,\,\,\,\,\, - \frac{4.9011}{{1 + e^{ - ( 13.757\theta - 27.113)} }} - \frac{0.0487}{{1 + e^{ - (1.2584\theta + 4.4397)} }}, \hfill \\ \end{gathered}$$

16 $$\begin{gathered} \hat{m}(\theta ) = \frac{ - 1.2311}{{1 + e^{ - (3.8031\theta - 3.0875)} }} - \frac{1.6094}{{1 + e^{ - ( - 7.3334\theta - 2.0239)} }} - \frac{2.2478}{{1 + e^{ - ( 2.5328\theta - 2.8606)} }} + \frac{2.1662}{{1 + e^{ - ( 5.0115\theta - 2.3467)} }} \hfill \\ \,\,\,\,\,\,\,\,\,\,\, - \frac{0.3374}{{1 + e^{ - ( - 6.173\theta - 2.5454)} }} - \frac{6.8904}{{1 + e^{ - ( - 7.844\theta - 12.2045)} }} + \frac{6.2610}{{1 + e^{ - ( - 7.642\theta - 2.7489)} }} - \frac{5.3609}{{1 + e^{ - ( 2.1072\theta - 6.2498)} }} \hfill \\ \,\,\,\,\,\,\,\,\,\,\,\, + \frac{5.0547}{{1 + e^{ - ( - 2.4580\theta - 3.7097)} }} + \frac{5.3493}{{1 + e^{ - ( - 1.9603\theta - 6.1331)} }}, \hfill \\ \end{gathered}$$

**Figure 2:**
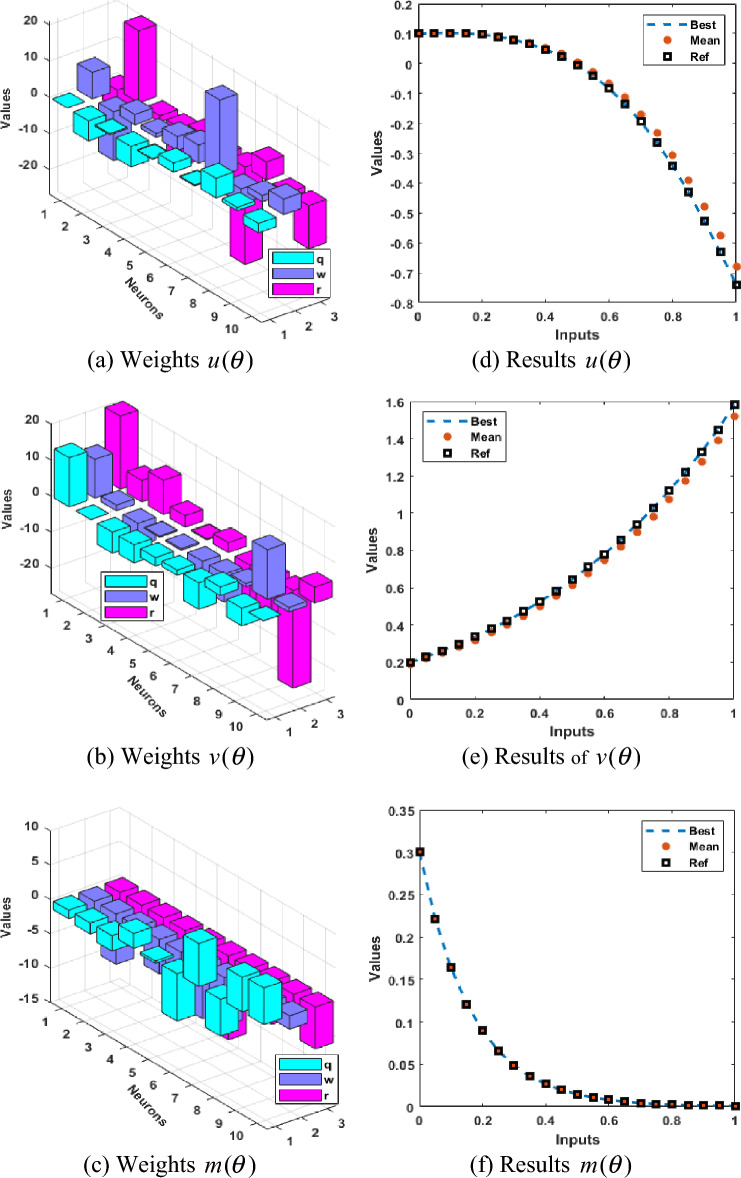
Weights and comparison of solution performances for the Rabinovich–Fabrikant system.

**Figure 3:**
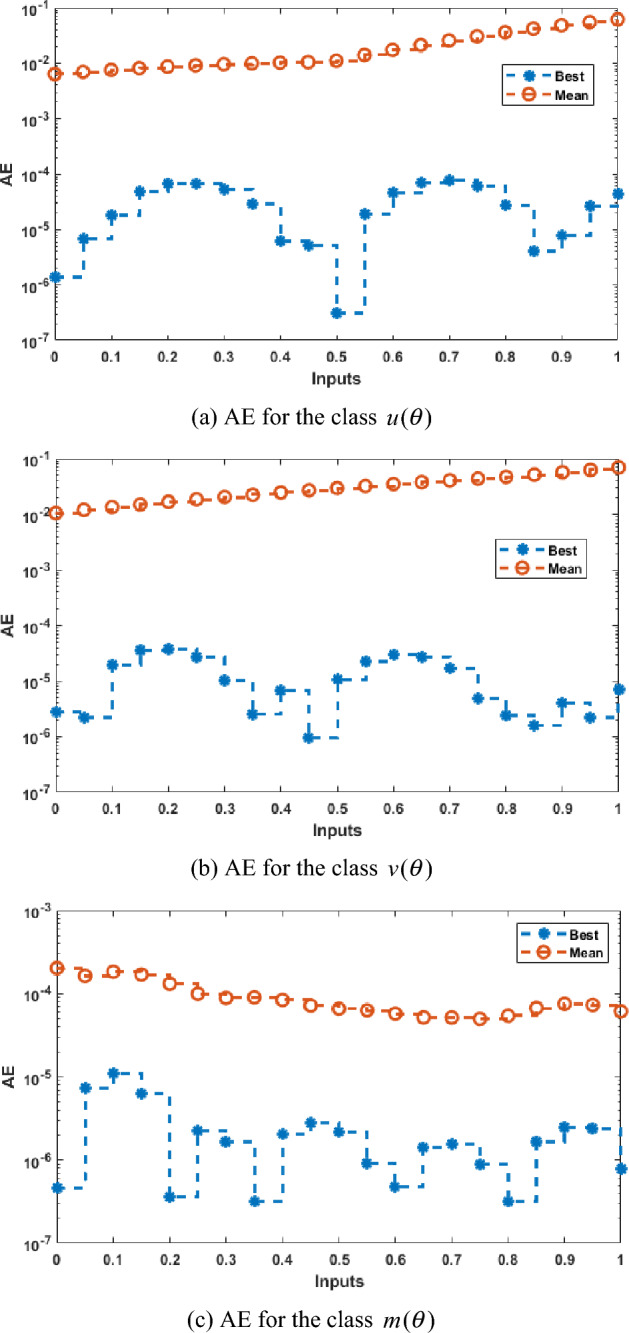
The values of AE to solve the Rabinovich–Fabrikant system.

**Figure 4:**
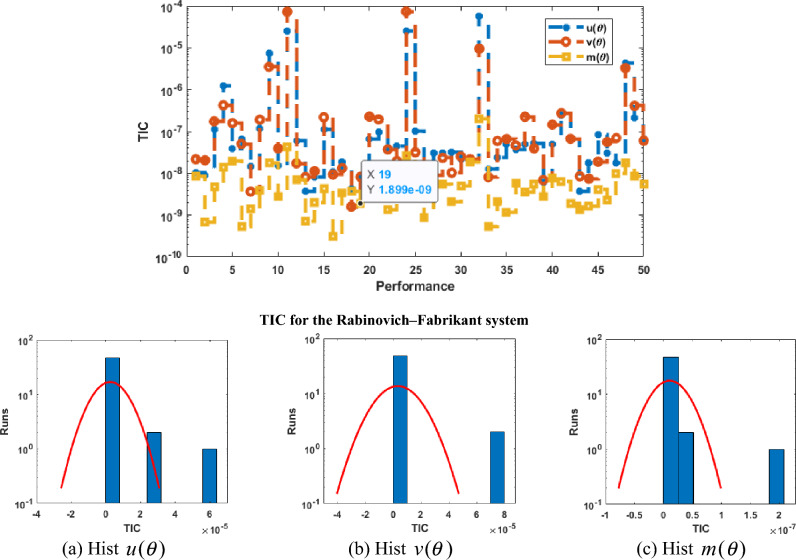
Performances of TIC along with hist values for the mathematical system.

**Figure 5:**
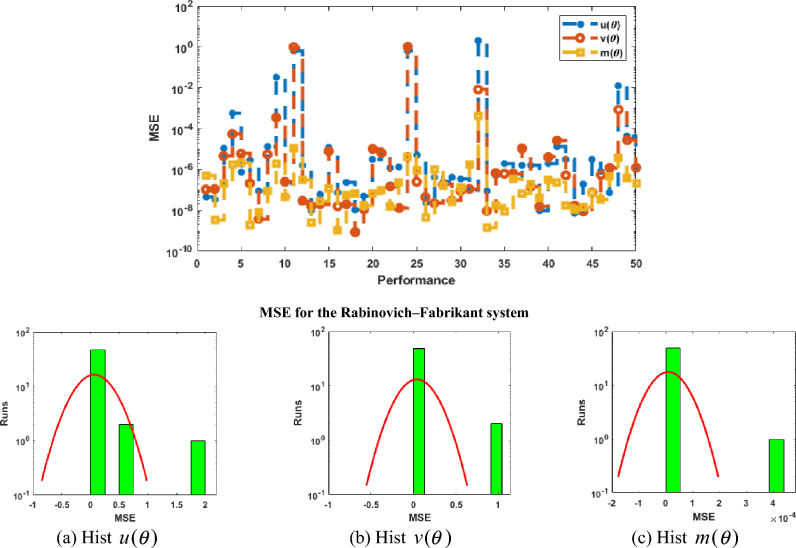
Performances of MSE along with hist values for the mathematical system.

Figure 2 illustrates the values of the optimal weights along with the result’s assessment for each class of the Rabinovich–Fabrikant system. These weights are shown in Fig. 2a–c by applying the ANNs together with the swarming computational approach to solve the Rabinovich–Fabrikant system by taking 10 numbers of neurons. The correctness of the ANNs together with the swarming and local computational method is examined through the best, reference and mean results in Fig. 2d–f. Figure 3 shows the mean and best results based on the AE to solve the Rabinovich–Fabrikant system. The best AE measures are performed as 10^−04^–10^−07^, 10^−05^–10^−07^ and 10^−06^–10^−07^, while the mean values of the AE are performed as 10^−02^–10^−03^, 10^−01^ to 10^−03^ and 10^−03^–10^−04^ for 1st–3rd dynamics. These reduceable performances of AE improve the precision of the ANNs. Figure 4 represents the performances of TIC that have been calculated based on the Rabinovich–Fabrikant system, which are found around 10^−06^–10^−10^ for each category. The MSE values are plotted in Fig. 5 to solve the Rabinovich–Fabrikant system through the stochastic approach. These performances are described as 10^−04^–10^−10^ for the system. The optimal statistical performances achieved through the ANNs together with the swarming scheme develop the method’s consistency to solve the above system.

Tables 1, 2, 3 shows the statistical operator measures for the minimum (best results), SIR, mean, maximum (worst outputs), median, and standard deviation (SD) values. The plots based on the maximum performances (bad results) reported as 10^−01^–10^−02^ for the first two dynamics of the model, while these values performed as 10^−03^–10^−04^ for the last dynamic of the model. The mean and SD operator values are 10^−02^–10^−03^ and 10^−01^–10^−02^ for the first two dynamics, while these values lie as 10^−04^–10^−05^ for the last dynamic of the model. Likewise, the median, minimum (best performances) and SIR operator values for each class of the Rabinovich–Fabrikant system are found as 10^−04^–10^−05^, 10^−06^–10^−07^ and 10^−03^–10^−05^. Based on these performances, the process of the ANNs together with the swarming and local search scheme perform precise to solve the Rabinovich–Fabrikant system.

**Table 1:** Different statistical measures for the Rabinovich–Fabrikant system (1).

$\theta$	$u(\theta )$					
	Maximum	Mean	Median	Minimum	SD	SIR
0	1.05690E−01	6.40790E−03	5.08468E−05	1.20218E−06	2.36816E−02	7.58571E−05
0.05	1.22841E−01	6.98814E−03	4.02634E−05	3.18951E−07	2.57846E−02	6.18554E−05
0.1	1.43050E−01	7.57637E−03	6.17875E−05	1.37938E−06	2.79626E−02	7.98119E−05
0.15	1.66086E−01	8.15105E−03	1.22617E−04	1.77658E−07	3.02299E−02	9.80690E−05
0.2	1.91909E−01	8.69372E−03	1.73347E−04	3.56693E−06	3.26824E−02	1.13967E−04
0.25	2.20686E−01	9.18423E−03	2.17530E−04	1.40996E−06	3.54503E−02	1.42554E−04
0.3	2.52773E−01	9.61410E−03	2.08632E−04	3.07849E−06	3.86938E−02	1.73721E−04
0.35	2.88683E−01	9.97919E−03	1.76354E−04	2.72037E−05	4.26073E−02	2.25364E−04
0.4	3.29034E−01	1.02867E−02	1.96363E−04	6.17216E−06	4.74130E−02	1.49917E−04
0.45	3.74495E−01	1.05397E−02	2.16955E−04	4.38184E−06	5.33532E−02	1.86919E−04
0.5	4.25732E−01	1.11452E−02	2.59888E−04	3.07209E−07	6.05991E−02	2.54918E−04
0.55	4.83354E−01	1.40952E−02	3.45508E−04	6.56680E−07	6.90186E−02	3.67314E−04
0.6	5.47875E−01	1.75133E−02	4.05529E−04	4.48314E−05	7.91777E−02	4.12311E−04
0.65	6.19674E−01	2.14188E−02	4.85687E−04	6.63475E−05	9.12644E−02	4.99647E−04
0.7	6.98962E−01	2.58265E−02	4.88451E−04	5.95039E−05	1.05424E−01	5.17867E−04
0.75	7.85725E−01	3.07458E−02	4.66624E−04	2.26947E−05	1.21745E−01	5.64742E−04
0.8	8.79659E−01	3.61768E−02	4.81259E−04	2.30813E−05	1.40240E−01	5.81885E−04
0.85	9.80067E−01	4.21008E−02	5.43547E−04	4.09488E−06	1.60816E−01	6.03728E−04
0.9	1.08574E−01	4.84540E−02	6.14579E−04	7.74713E−06	1.83256E−01	7.85763E−04
0.95	1.19485E−01	5.51122E−02	7.79590E−04	1.55711E−06	2.07173E−01	6.99057E−04
1	1.30480E−01	6.17905E−02	8.51394E−04	1.89442E−06	2.32010E−01	7.90777E−04

**Table 2:** Different statistical measures for the Rabinovich–Fabrikant system (2).

$\theta$	$v(\theta )$					
	Maximum	Mean	Median	Minimum	SD	SIR
0	1.80930E−01	1.05117E−02	1.56302E−04	1.20218E−06	3.85499E−02	2.28766E−04
0.05	2.16171E−01	1.20503E−02	1.78333E−04	3.18951E−07	4.49069E−02	2.85149E−04
0.1	2.49110E−01	1.35299E−02	1.75805E−04	1.37938E−06	5.12729E−02	3.03159E−04
0.15	2.84048E−01	1.50777E−02	1.89514E−04	1.77658E−07	5.80619E−02	3.11228E−04
0.2	3.23084E−01	1.67597E−02	2.18140E−04	3.56693E−06	6.55171E−02	3.24209E−04
0.25	3.65827E−01	1.85756E−02	2.48140E−04	1.40996E−06	7.36480E−02	4.01238E−04
0.3	4.12074E−01	2.05200E−02	2.79358E−04	3.07849E−06	8.24701E−02	4.84476E−04
0.35	4.61942E−01	2.25962E−02	2.83904E−04	2.72037E−05	9.20213E−02	5.49853E−04
0.4	5.15676E−01	2.48089E−02	2.86481E−04	6.17216E−06	1.02348E−01	6.16102E−04
0.45	5.73546E−01	2.71609E−02	3.19916E−04	4.38184E−06	1.13500E−01	5.65715E−04
0.5	6.35804E−01	2.96547E−02	2.79361E−04	3.07209E−07	1.25519E−01	6.06080E−04
0.55	7.02671E−01	3.23225E−02	3.10564E−04	6.56680E−07	1.38442E−01	8.43722E−04
0.6	7.74346E−01	3.51264E−02	3.57440E−04	4.48314E−05	1.52317E−01	9.45295E−04
0.65	8.51024E−01	3.80537E−02	4.28832E−04	6.63475E−05	1.67193E−01	1.03409E−03
0.7	9.32931E−01	4.10722E−02	5.16832E−04	5.95039E−05	1.83134E−01	1.12184E−03
0.75	1.02040E−01	4.41658E−02	5.67579E−04	2.26947E−05	2.00230E−01	1.20797E−03
0.8	1.11397E−01	4.73450E−02	5.75595E−04	2.30813E−05	2.18615E−01	1.26351E−03
0.85	1.21454E−01	5.17237E−02	5.70407E−04	4.09488E−06	2.38257E−01	1.33663E−03
0.9	1.32354E−01	5.70127E−02	5.87427E−04	7.74713E−06	2.59548E−01	1.46495E−03
0.95	1.44317E−01	6.29763E−02	6.95371E−04	1.55711E−06	2.83011E−01	1.72981E−03
1	1.57650E−01	6.97606E−02	8.67320E−04	1.89442E−06	3.09254E−01	1.98607E−03

**Table 3:** Different statistical measures for the Rabinovich–Fabrikant system (3).

$\theta$	$m(\theta )$					
	Maximum	Mean	Median	Minimum	SD	SIR
0	6.58629E−03	2.02783E−04	1.55765E−05	1.20218E−06	9.33476E−04	2.82452E−05
0.05	5.01335E−03	1.63321E−04	3.64540E−05	3.18951E−07	7.04530E−04	3.23278E−05
0.1	3.95453E−03	1.83999E−04	5.27212E−05	1.37938E−06	5.58475E−04	5.83354E−05
0.15	3.17549E−03	1.69807E−04	5.08574E−05	1.77658E−07	4.57572E−04	6.92912E−05
0.2	2.56724E−03	1.31963E−04	2.40379E−05	3.56693E−06	3.77014E−04	4.77252E−05
0.25	2.07883E−03	9.98185E−05	1.84659E−05	1.40996E−06	3.10152E−04	3.22076E−05
0.3	1.68442E−03	8.91099E−05	3.09639E−05	3.07849E−06	2.56272E−04	2.17263E−05
0.35	1.36831E−03	9.03436E−05	3.79007E−05	2.72037E−05	2.13215E−04	2.64122E−05
0.4	1.11868E−03	8.42805E−05	2.71341E−05	6.17216E−06	1.80472E−04	3.13689E−05
0.45	9.25173E−04	7.17091E−05	2.33858E−05	4.38184E−06	1.54250E−04	2.63757E−05
0.5	7.77852E−04	6.60226E−05	2.41276E−05	3.07209E−07	1.28257E−04	2.95578E−05
0.55	6.68033E−04	6.31421E−05	3.93428E−05	6.56680E−07	1.05745E−04	2.73639E−05
0.6	5.87681E−04	5.76348E−05	3.32827E−05	4.48314E−05	9.08890E−05	2.44285E−05
0.65	5.29980E−04	5.17476E−05	2.58827E−05	6.63475E−05	8.24802E−05	2.16873E−05
0.7	4.89313E−04	5.16222E−05	3.40469E−05	5.95039E−05	7.67325E−05	2.12499E−05
0.75	4.61136E−04	4.97995E−05	2.54209E−05	2.26947E−05	7.96171E−05	1.61246E−05
0.8	4.41978E−04	5.43080E−05	1.88782E−05	2.30813E−05	8.53107E−05	2.74754E−05
0.85	4.29426E−04	6.73073E−05	3.04463E−05	4.09488E−06	8.79037E−05	3.25790E−05
0.9	4.21844E−04	7.54484E−05	4.33264E−05	7.74713E−06	9.04288E−05	3.38840E−05
0.95	4.18417E−04	7.30378E−05	4.63993E−05	1.55711E−06	9.43253E−05	2.54015E−05
1	4.19007E−04	6.14300E−05	1.74213E−05	1.89442E−06	1.04388E−04	2.89049E−05

## Conclusions

The current investigations present a stochastic computing reliable scheme based on the swarming computing procedure for the numerical solutions of the Rabinovich–Fabrikant system. The system’s dynamic of the nonlinear system has three coupled equations. Some of the concluding remarks are itemized as:

- The computing stochastic artificial neural networks along with the global swarming and local search interior point algorithms have been presented to solve the differential form of the Rabinovich–Fabrikant system.
- The design of objective function has been presented by using the differential system, while the optimization is performed through the local and global search schemes.
- The accuracy of the results has been observed through the achieved and source results performances.
- The log-sigmoid transfer function along with 10 numbers of neurons in the structure of neural network have been provided for the solutions of the Rabinovich–Fabrikant system.
- The absolute error has also been achieved around 10^−05^–10^−08^, which shows the worth of the ANNs-PSOIP algorithm.
- The consistency of the ANNs-PSOIP method has been examined by applying different statistical performances to solve the Rabinovich–Fabrikant system.

In future, the designed ANNs along with the swarming scheme is provided to perform the solutions of the biological system^67,68^, and fluid dynamical systems^69^.

## Data Availability

The datasets generated/produced during and/or analyzed during the current study/research are available from the corresponding author on reasonable request.
